# Double Layer Reconstruction of Exposed Cardiac Implantable Electronic Devices in Elderly Patients

**DOI:** 10.7759/cureus.13024

**Published:** 2021-01-30

**Authors:** Burak Ozkan, Abbas Albayati, Kerem C Yilmaz, Orcun Ciftci, Bulent Ozin, Cagri A Uysal, Nilgun Markal Ertas

**Affiliations:** 1 Plastic and Reconstructive Surgery, Baskent University Faculty of Medicine, Ankara, TUR; 2 Cardiology, Baskent University Faculty of Medicine, Ankara, TUR

**Keywords:** exposed cardiac implants, cardiac implantable electronic device exposition, pectoralis major muscle flap, dual layer closure, double layer reconstruction, breast flap, pacemaker exposition

## Abstract

Background

Elderly patients with multiple comorbidities may not be candidates for cardiac implanted electronic device (CIED) explantation in cases of exposition. Excision of all unhealthy and inflamed scar tissue results in a skin defect that must be covered. Small- to moderate-sized local skin flaps and subpectoral placement of CIEDs have been described in the literature. However, these techniques still could not eliminate the risk of recurrence. In terms of minimizing the recurrence risk, we aim to increase the flap dimensions for getting better circulation and tension-free closure after subpectoral placement.

Material and methods

Six patients who were operated for a dual-layer reconstruction of exposed cardiac implants between 2017 and 2020 were included in the study. All patients were referred to plastic surgery as soon as the wound biopsy culture results were negative after systemic and topical antibiotic treatment by cardiology department.

Results

No flap loss or wound dehiscence was seen with a mean duration of 11 months follow-up. Early hematoma was encountered in a patient who was managed with irrigation and drain renewal. One patient developed suture abscess in the second month postoperatively. Knots were removed and wound healed without further intervention.

Conclusion

Double layer closure of exposed cardiac implants with large breast fasciocutaneous flap after subpectoral placement of pulse generator and leads suggest durable and reliable coverage in elderly patients with multiple comorbidities.

## Introduction

Cardiac implantable electronic devices (CIEDs) have been used for the management of a wide range of cardiac problems, such as bradycardia and the regulation of heart contractibility in congestive heart failure, for over 50 years [1]. Dealing with CIED-related complications, such as infection, skin necrosis, wound dehiscence, exposition, implant erosion, and infection, has been a challenge since its first application [2]. There are various suggested treatment modalities in the literature, including topical wound care and systemic antibiotic regimes [3, 4]. However, the conservative approach is generally not sufficient when the CIED is exposed [5]. Extracting the CIED with leads and changing the implantation site is a widely accepted treatment [6]. Nevertheless, lead extraction may be a challenging procedure in a high-risk patient population such as the elderly with many comorbidities [7]. In this patient group, salvaging of the CIEDs is managed by dealing with bacteremia using topical/systemic antibiotics and flap coverage [8]. The literature reports various reconstructive methods, including single- or double-layer closure with small- to moderate-sized local skin flaps and pectoralis major muscle. These methods have different recurrence and success rates [9, 10, 11]. We aimed to decrease the recurrence risk by repositioning the leads of the CIED under the pectoralis major muscle and increasing the skin flap dimensions.

In this retrospective study, we present the outcomes of our double-layer closure method for salvaging exposed CIED in elderly patients using the pectoralis muscle and large fasciocutaneous breast flaps.

## Materials and methods

This study included six patients older than 60 years of age operated on for eroded cardiac implants in the Baskent University Plastic and Reconstructive Surgery Department between 2017 and 2020. All patients were referred from the cardiology department to cover the existing pacemaker system. Before the plastic surgery consultation, the cardiology department administered systemic and topical antibiotic treatments to the patients. Wound swap and tissue biopsies were negative in all patients. All the anticoagulant therapies stopped and low molecular weight heparin treatment started five days prior to the surgery. Anti-tachycardia therapy was switched off in the implantable cardiac defibrillator devices, and stable pace modes were programmed in pace-dependent patients before the surgery. The electrophysiology team joined the operation for guidance and need for any urgent intervention in case of any lead violation. Age, gender, American Society of Anesthesiologists (ASA) scores, comorbidities, the duration of implant, the duration of exposition, operation time, complications, and follow-up time were noted. 

Surgical technique

**Figure 1:**
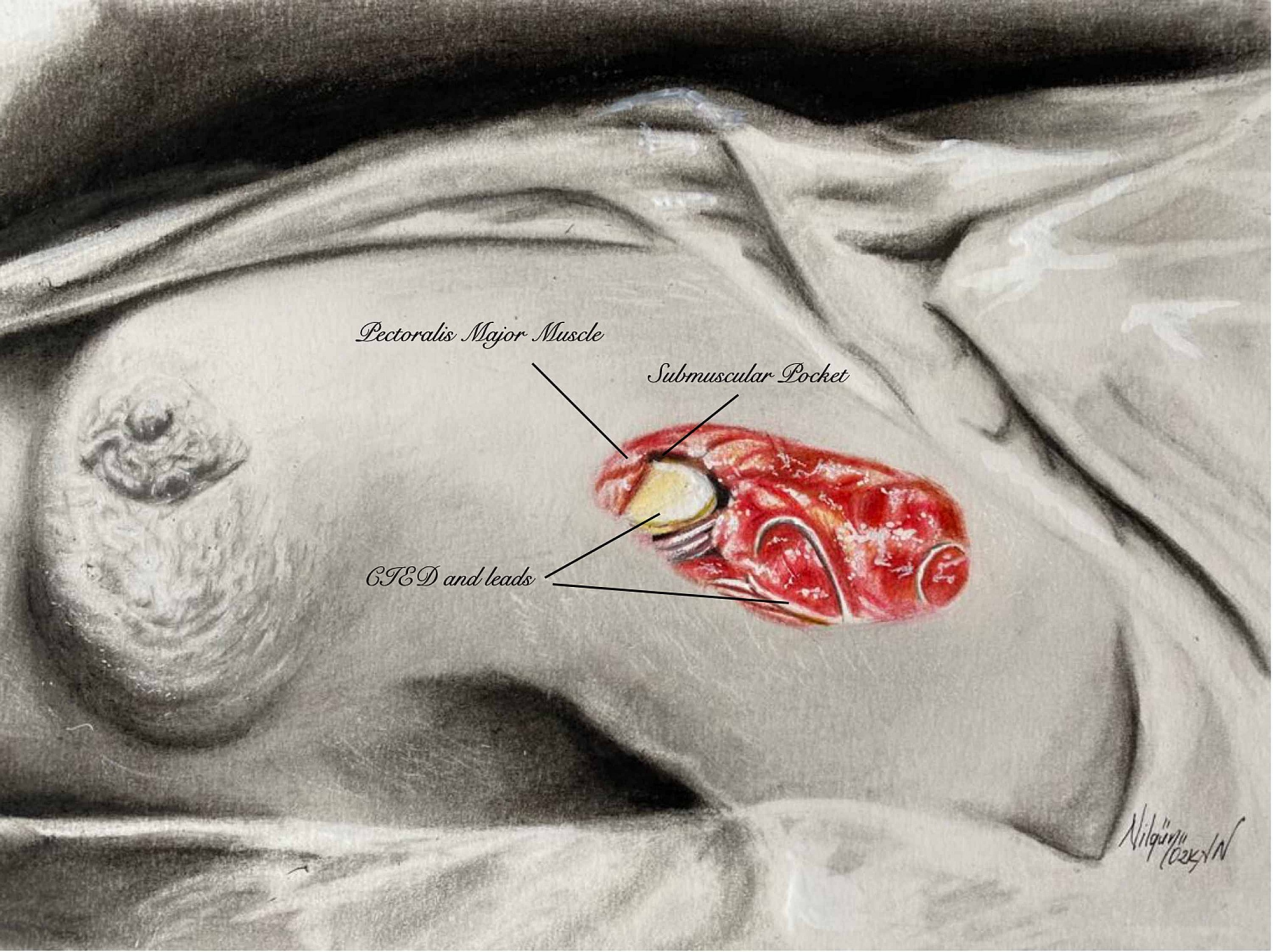
Illustration of the placement of CIED under the subpectoral pocket after hockey stick (J shaped) incision CIED: cardiac implantable electronic devices

All operations were performed under deep sedation. Patients lay on the operation table in a supine position with arms abducted to 90 degrees. A careful debridement of existing scar tissue was done. The leads surrounded by fibrous capsules were carefully dissected and liberated to avoid a cut or injury. After the CIED and leads were irrigated with saline and povidone-iodine solutions, lidocaine was injected into the pectoralis major fascia for further pain control. A transverse incision was made parallel to the pectoralis major fibers. A blunt dissection was performed under the pectoralis major muscle, and a submuscular pocket slightly larger than the implant size was prepared to accommodate leads and device (Figure 1). 

**Figure 2:**
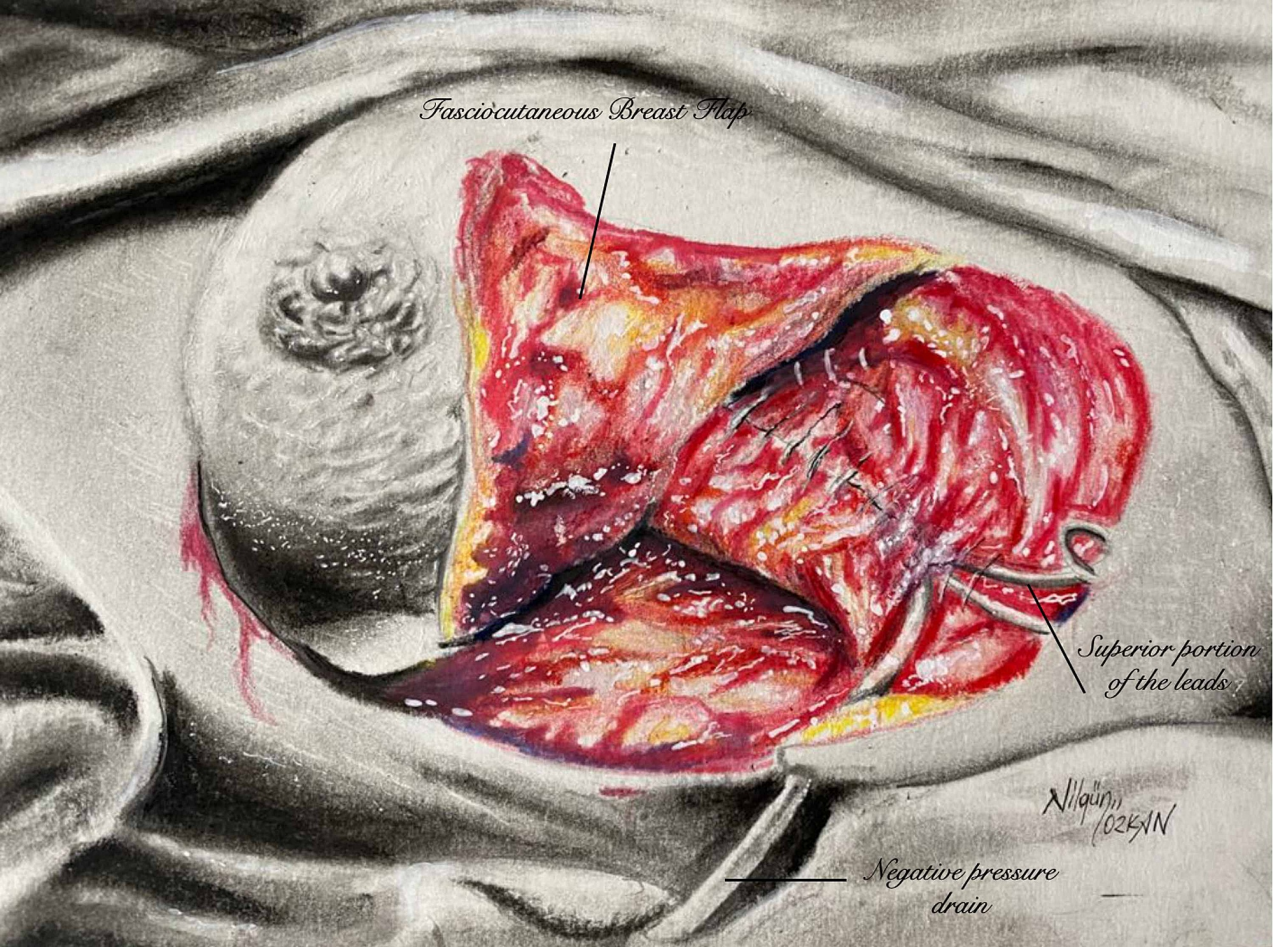
Illustration of elevated large fasciocutaneous pectoral skin flap which makes the second layer of closure. Tip of the flap was de-epithelized and sutured over superior part of the leads.

The rest of the leads were placed under the muscle. The pectoralis major muscle was separated superiorly with bipolar cautery up to the subclavicular portion of the leads. A rectangular skin flap was marked along the anterior axillary border laterally and inframammary sulcus inferiorly, and a back cut was made to the midsternal line medially if needed (Figure 2).

The flap was elevated from the pectoralis major fascia after local anesthetic injection. Perforators from the pectoralis major muscle were preserved if they were not restricting rotation advancement. The tip of the flap was de-epithelized and advanced subcutaneously over the subclavicular portion of the leads, which could not be buried under the muscle. Two negative pressure drains were placed under the skin flap, and one drain was placed under the muscle for hematoma prevention.

## Results

The mean age of the patients was 75 years (65-85). The mean duration of the operation was 116 minutes. Patients were hospitalized and monitored in the cardiac intensive care unit for two days postoperatively. No flap necrosis or dehiscence was observed. Hematoma developed under the skin flap on the second postoperative day in one patient. Subcutaneous pockets with irrigated drains were placed. The patient healed uneventfully. There were no late complications except for a suture abscess in one patient during the mean follow-up period of 11 months. Suture abscesses were encountered two months after the operation. The 2.0 Vicryl (Ethicon, Inc., Bridgewater, NJ, USA) knot was removed from the discharge site, and the patient healed with secondary intention. Patients did not complain about any restriction, discomfort, or pain during shoulder abduction. Table 1 presents the demographics and clinical features of the patients. 

**Table 1:** The demographic and the clinical characteristics of the study population ASA: American Society of Anesthesiologists

Patients	Gender	Age	Type of The Device	Duration of Cardiac Device	Duration of Exposition	Comorbidities	ASA Score	Operative Time	Complications	Follow-up
Patient 1	Male	66	Pacer (Medtronic)	8 years	1 month	Aortic valve replacement, atrioventricular block	III	100 minutes	Early hematoma	14 months
Patient 2	Male	80	Cardiac Resysnchronization therapy (CRT-D/Medtronic)	7 years	2 months	Chronic renal disease, coronary artery disease, atrial fibrillation	IV	150 minutes	Suture abscess	8 months
Patient 3	Male	72	Pacer (Medtronic)	3 years	5 days	Chronic renal disease, diabetes mellitus, atrial fibrillation	III	100 minutes	-	6 months
Patient 4	Female	85	Cardiac Resysnchronization therapy (CRT-D/Medtronic)	3 years	Skin necrosis without exposition	Heart failure, atrial fibrillation	IV	120 minutes	-	6 months
Patient 5	Male	65	Pace (Indengio/Boston Scientific)	3 years	5 days	Coronary artery disease, hypertension, chronic obstructive lung disease	IV	100 minutes	-	22 months
Patient 6	Male	82	Implanted cardiac defibrillator (Medtronic)	9 years	10 days	Coronay artery disease, congestive heart disease, hypertension D	IV	130 minutes	-	10 months

Case examples 

Case 1

**Figure 3:**
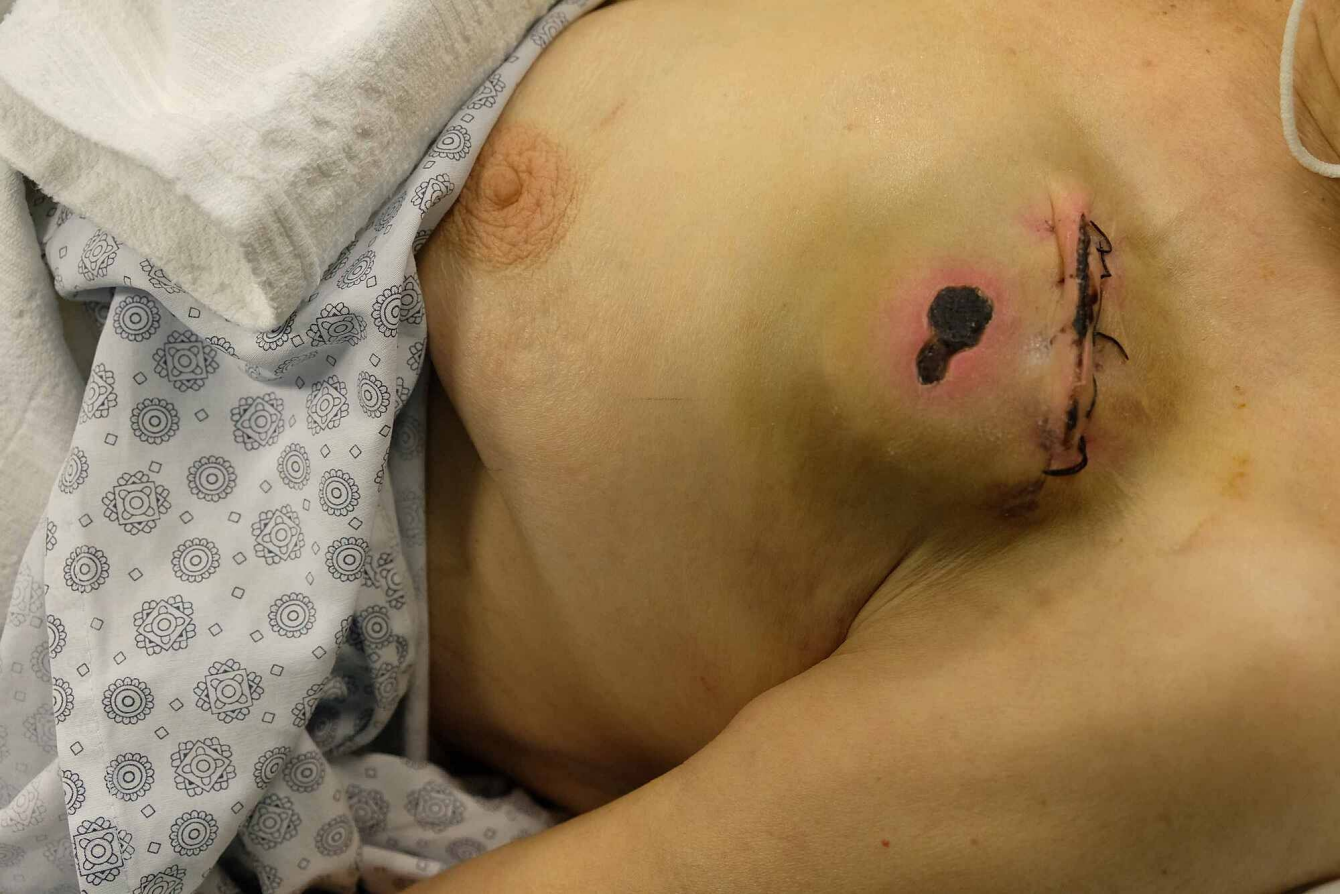
The patient had skin necrosis over implantation site and hematoma formation around the implanted device.

The cardiology department referred an 85-year-old female patient for skin necrosis over her pacemaker. She had decompensated heart insufficiency, and the cardiac pacemaker that she had for three years needed a replacement one month ago. The skin over the pacemaker site had 2x1 cm demarcated skin necrosis and perinecrotic inflammation (Figure 3).

**Figure 4:**
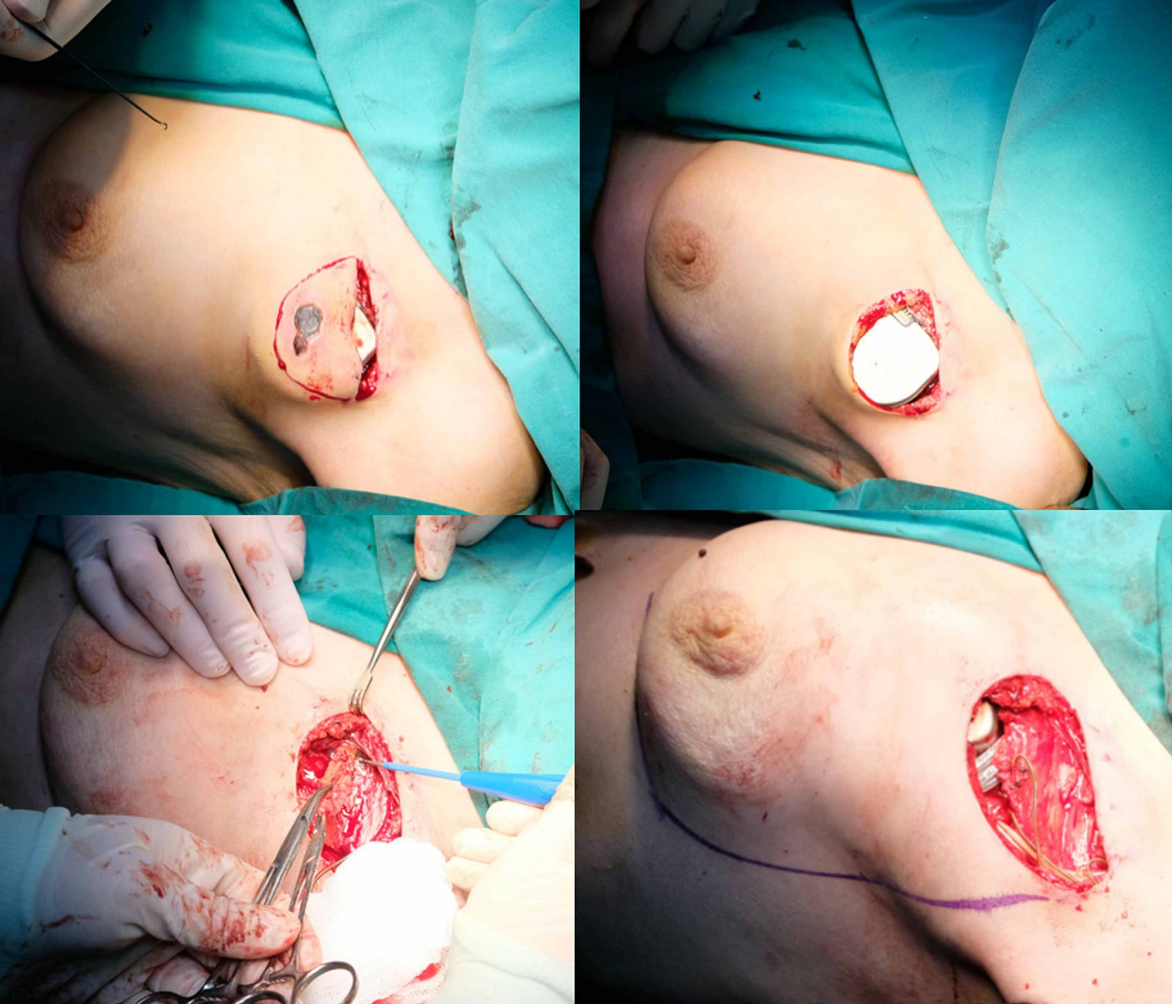
(Top) All the inflamed, unhealthy tissues were excised; (bottom left) capsule formation around the device was also derided over the pectoralis muscle; (bottom right) flap borders were marked after placement of CIED under pectoralis major muscle CIED: cardiac implantable electronic device

Ultrasonographic examination showed hematoma formation around the implant. A decision was made to cover the implant with dual-layer healthy tissue before the spontaneous exposition. Anticoagulating therapy was halted, and low molecular weight heparin treatment (LMWH) was started five days prior to surgery. The operation was performed under deep sedation and concomitant local anesthesia with prilocaine. All necrotic and hyperemic skin was excised. Capsule formation was debrided carefully with the supervision of the attending cardiologist. A 3-cm-long transverse incision was made on the pectoralis major muscle, and a blunt dissection was made under the pectoralis muscle to prepare a submuscular space for the device (Figure 4).

**Figure 5:**
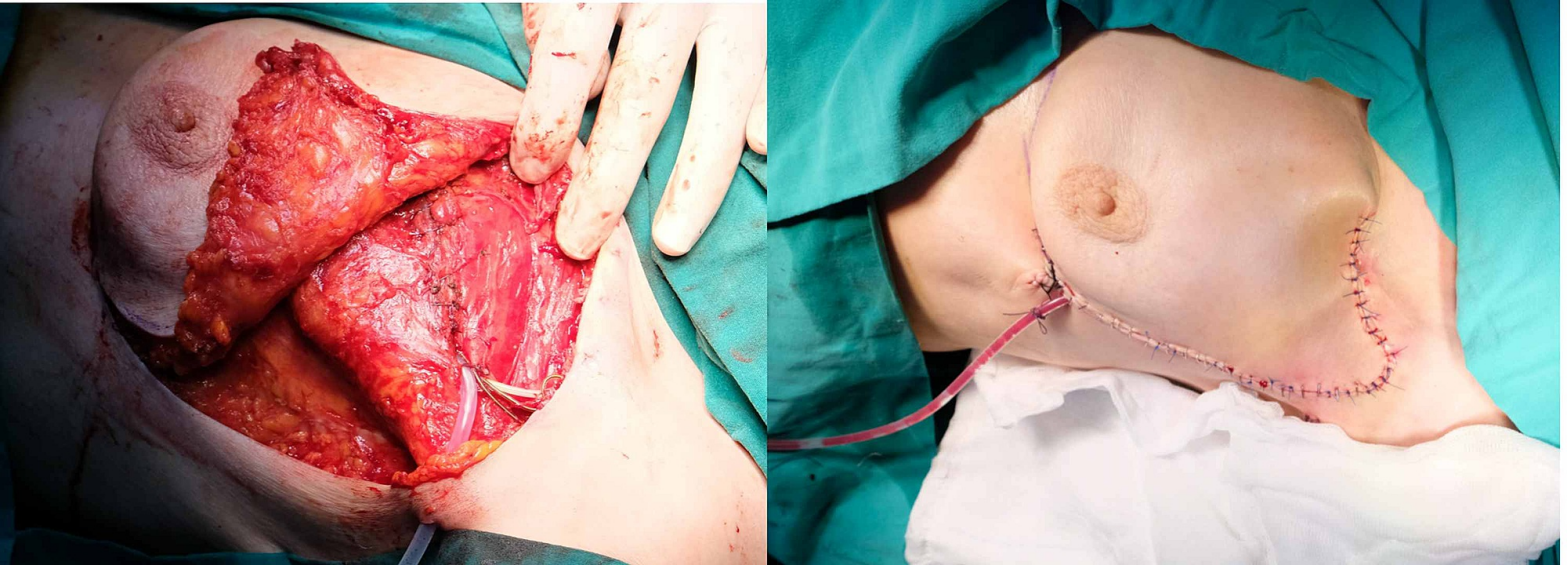
(Left) under the elevated fasciocutaneous breast flap, muscle was closed with 2.0 Vicryl; (right) immediate image of the patient after reconstruction

After placement of the device and the lead, a superior oblique incision was made with help of bipolar cautery to pectoralis major to accommodate the wires in the subclavicular region. Then a rectangular-shaped breast skin flap was elevated for tension-free closure of the defect. The tip of the flap was de-epithelized and sutured over the last visible part of the wires. Drains were placed under subcutaneous and submuscular pockets (Figure 5).

**Figure 6:**
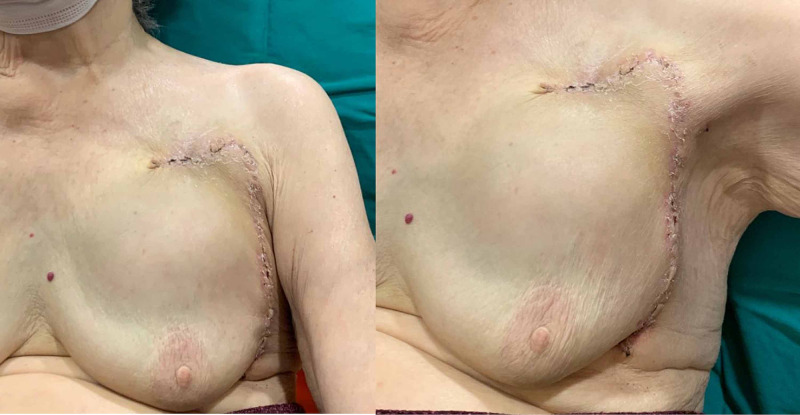
Postoperative second-month image of the patient; patient did not complain of any restriction or discomfort during shoulder movements

A closed incisional negative pressure wound therapy (ciNPWT) was started immediately after the suturation of the operation site. Drains were removed on the fifth postoperative day. CiNPWT was applied for three sessions. No flap necrosis or dehiscence was encountered. Figure 6 demonstrates the postoperative second-month image of the patient. 

Case 2

**Figure 7:**
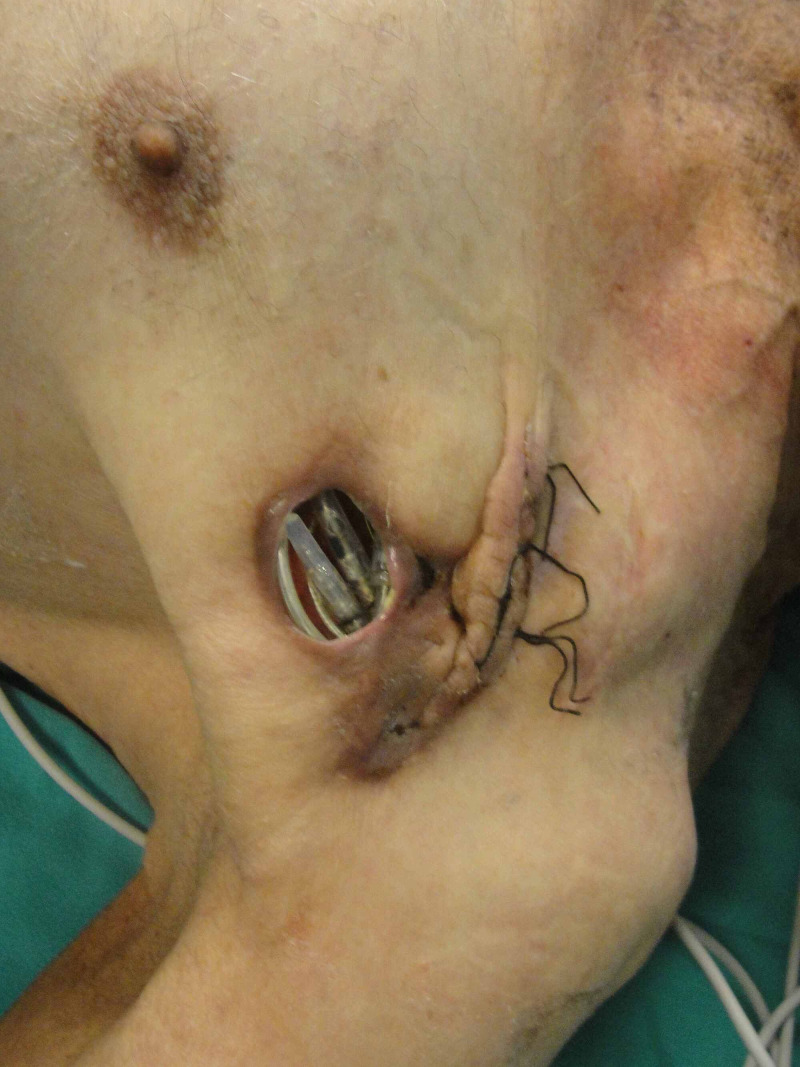
Pre-operative view of the exposed CIED CIED: cardiac implantable electronic devices

An 80-year-old male patient was referred to the plastic surgery department for an exposed cardiac resynchronization therapy device (CRT-D). His medical history showed chronic renal failure, two coronary artery bypass surgeries, and a CRT-D placement seven years ago. He underwent CRT-D battery replacement a year ago. He was hospitalized for 20 days due to cellulite formation at the operation site, which started two months after the replacement. The patient encountered implant exposition in the subsequent months. The primary closure attempts by the cardiology department failed and resulted in a 4x4 cm sized defect with the total exposition of the CRT-D device (Figure 7). 

**Figure 8:**
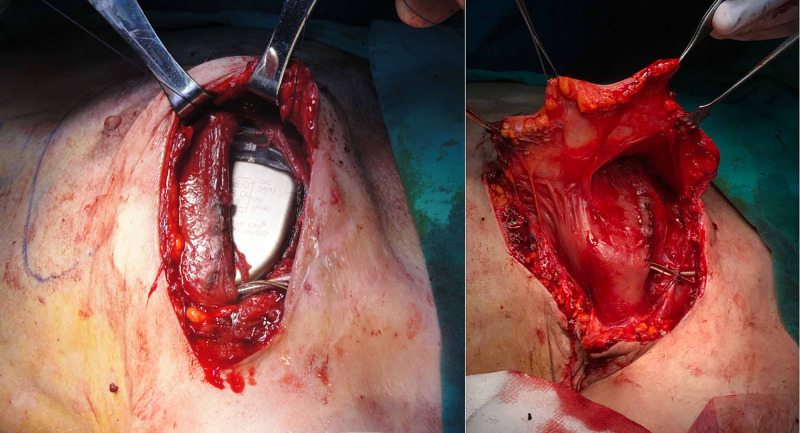
(Left) subpectoral placement of the CIED; (right) elevation of large fasciocutaneous breast flap for coverage of superior portion of the leads and skin defect CIED: cardiac implantable electronic devices

Because of the numerous comorbidities of the patient, the extraction of the implant was not recommended, and a dual-layer flap reconstruction was planned for the patient. Anticoagulants were stopped, and LMWH was started. A temporary pace was placed prior to surgery. After adequate debridement of the necrotic tissues and capsule around the device, a hockey stick incision (3-cm-long transverse, 4 cm superior oblique) was made on the pectoralis major muscle. The device and leads were placed under the bluntly dissected submuscular plane. After the closure of the muscle with the 2.0 round tip Vicryl suture, a 10x7 cm rectangular breast flap was raised over the pectoralis muscle fascia to close the skin defect (Figure 8).

**Figure 9:**
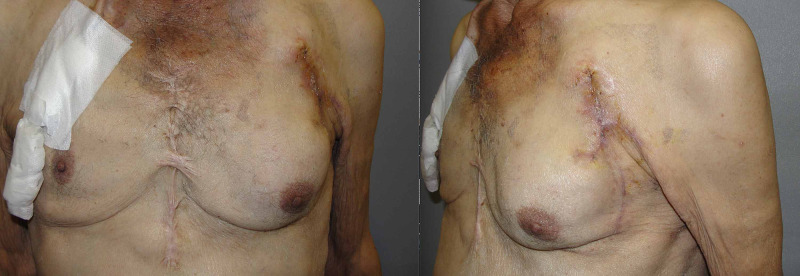
Postoperative third month view of the patient

The flap tip was de-epithelized and adapted over the remnant part of the CRT-D leads. Two negative-pressure suction drains were placed on the submuscular and subdermal planes. Follow-up visits indicated no early- or late-term complications. Figure 9 demonstrates the late postoperative images of the patient

## Discussion

Urgent intervention should be performed once the CIED is exposed. Although lead extraction and placing it on the contralateral site is the desired option, the general condition of the patient may hinder the conservative decision of CIED explantation [12]. CIED explantation carries complication risks, such as cardiac and vascular avulsions requiring thoracotomy [13]. In this patient group, the reasons for the exposition should be well considered before planning the reconstruction.

In our patient group, most of the patients were over 80 years old. Elderly patients have skin characteristics, such as decreased subdermal fat and dermal thickness, which lead to exposition. All of our patients had more than two years of CIED history. All of them had peri-implant capsules around the device. There has been an increasing number of publications supporting the role of biofilm in device-related infections [14]. This biofilm layer aggravates capsule formation [15]. The capsule causes skin contraction and shrinkage, which might increase the risk of exposition [16]. Sixty-six percent of our patients had a history of pulse generator battery replacement. It is well known that concurrent incisions at the same operation site increase the risk of wound complications [17]. Therefore, we excised all the scarred and inflamed tissue along with the capsule during debridement. 

The superior pectoral or anterolateral thorax region has potential for reconstructive options. Pectoralis major muscle is one of the most favored tissues for covering CIEDs [18]. The submuscular placement of cardioverter defibrillators has aesthetic superiority, especially in the younger population [19]. In our study, all the CIEDs were placed via the transverse pectoral approach. Therefore, we used the existing defect to approach the pectoralis muscle. Any position change was needed such as a latissimus dorsi flap elevation, which is an alternative muscle option in the region [20]. The pectoralis major muscle has a robust vascular circulation that provides safe accommodation of the implant and its leads. We made a hockey stick incision with bipolar cautery to reach the submuscular plane. During this step, meticulous care should be taken to avoid violation of the dominant pedicle of the muscle, which lies between the midclavicular line and the distal third lateral of the clavicular line. Pectoralis major flap utilization was criticized by authors for its discomfort during shoulder movements and risk for hematoma formation [21]. However, we did not encounter any complaints about restricted abduction in the follow-up. We restricted shoulder movements with a Velpau bandage for better healing and pain control for a two-week postoperative period. Toia et al. stated that pectoralis major discomfort decreased with time in their patients, similar to our experience [22]. 

Various flaps are recommended for covering skin defects after CIED exposition. Existing literature mostly describes limited-sized local transposition flaps, such as Limberg flaps or rotation flaps [11]. However, the risk of recurrence due to persistent infections caused by bacterial inoculation to the device should be considered [23]. We thought that a small local flap for covering the device would not provide adequate tension-free closure. Additionally, another site for flap elevation would be needed in cases with recurrence. We aimed to elevate a large fasciocutaneous breast flap over the pectoralis major muscle to overcome these drawbacks. This flap provided healthy tissue to the exposition site and had reliable perfusion based on intercostal perforators. The advantages of this flap were reliable perfusion, closure without tension, limited scarring along breast borders, minimal donor site morbidity, and a chance for re-elevation. The flap could be raised under deep sedation with local anesthesia. There were some disadvantages to this technique. First, the operative time was slightly higher compared with other methods. Second, although incisions were around the breast border and the nipple-areolar complex was not violated, the surgery caused postoperatively acceptable breast asymmetry. Third, the dissection area was larger than that of small flaps, and meticulous hemostasis and drain placement were needed for hematoma prevention. We additionally advise closed incisional negative pressure application for the prevention of dehiscence, hematoma, and edema relief at the operation site for a one-week postoperative period [24]. 

To summarize, our dual-layer reconstruction consisted of covering the CIED and its leads with the pectoralis muscle and a large fasciocutaneous breast flap. This technique decreased the recurrence rate and the risk of exposition in elderly patients with multiple comorbidities. Large patient samples and longer follow-up periods are required for better evaluation of the outcomes.

## Conclusions

Reliable coverage of the cardiac implants and leads is essential in patients who are not candidates for CIED extraction. Our results suggest that reliable and durable coverage of the exposed cardiac implants can be achieved by a dual-layer reconstruction method with submuscular placement and a large fasciocutaneous breast flap.
